# Perception of medical and nursing students towards interprofessional nurse-physician collaboration at Al-Azhar University: a comparative cross-sectional study

**DOI:** 10.1186/s12909-025-07150-6

**Published:** 2025-05-05

**Authors:** Doaa Sadek Ahmed, Rania Hassan Hassan, Doaa Ali Soliman Ali, Safa Hamdy Alkalash, Abeer A. Almowafy, Samar Samy Ismail

**Affiliations:** 1 Community and Occupational Medicine Department, Faculty of Medicine for Girls, Al-Azhar University, Cairo, Egypt; 2 Technical Institute of Nursing, Faculty of Medicine for Girls, Al-Azhar University, Cairo, Egypt; 3 Department of Community Medicine and Healthcare, Faculty of Medicine, Umm Al-Qura University, Al-Qunfudha, Saudi Arabia; 4 International Islamic Institute for Population Studies and Research, Al-Azhar University, Cairo, Egypt

**Keywords:** Interprofessional collaboration, Perception, Nursing students, Medical students

## Abstract

**Background:**

Effective healthcare relies on strong collaboration between nurses and physicians. Interprofessional education [IPE] plays a vital role in fostering positive attitudes of medical and nursing students toward this collaboration. This study aims to explore the perceptions of nurse-physician collaboration among medical and nursing students at Al-Azhar University in Egypt, along with its related factors.

**Methods:**

This cross-sectional study included 364 female medical and nursing students. Data were collected using a self-administered questionnaire comprising socio-demographic and academic information, along with the validated Jefferson Scale of Attitudes toward Physician–Nurse Collaboration (JSAPNC), which assesses attitudes toward nurse-physician collaboration.

**Results:**

Among the participants, there were 153 medical students (including 25 house officers) and 211 nursing students (including 20 interns). Nursing students demonstrated higher scores across all subdomains of the JSAPNC, with a significantly higher total median score (47.9 vs. 44.8, *p* < 0.05). Nursing students with a positive perception had significantly lower parental education levels and family income compared to medical students (p-value < 0.05). Additionally, 55.7% of nursing students had a history of clinical training, compared to only 9.3% of medical students (p-value < 0.001). The determinants of positive perception among students differed, with 53.3% of nursing students citing clinical observations in hospitals, while 76.7% of medical students identified social media as the primary factor. Moreover, medical students exhibited a more positive overall attitude towards nurse-physician collaboration than house officers, with a total score median of 44 vs. 42 (p value = 0.05). However, no significant differences were observed between nursing students and interns across all JSAPNC domains.

**Conclusion:**

Nursing students had a more positive attitude toward nurse-physician collaboration than medical students; their perceptions were shaped by clinical training, while social media impacted medical students. Enhancing structured teamwork in training and addressing social media roles can improve collaboration and patient care.

**Supplementary Information:**

The online version contains supplementary material available at 10.1186/s12909-025-07150-6.

## Introduction

The interaction between nursing and medical students is critical as these future practitioners will collaborate closely in clinical settings. Exploring their perceptions of collaboration is essential, as positive attitudes can foster improved teamwork, effective communication, and enhanced patient care [1].

Strong nurse-physician partnerships not only improve patient satisfaction but also reduce errors and increase healthcare efficiency, underscoring the vital role of interdisciplinary collaboration in achieving high-quality outcomes [2]. Conversely, misunderstandings and lack of collaboration can hinder patient care and create barriers in the healthcare environment. Studies reveal that negative perceptions of interprofessional collaboration (IPC) can stem from historical hierarchies, communication barriers, and differing professional cultures [3].

Physicians and nurses have distinct roles in everyday healthcare settings, they do not have the same leadership experience as they have different educational backgrounds and different scopes of daily practice and hold different positions towards patients. Physicians typically adopt a more authoritative role in diagnosing and treating medical conditions, while nurses, with their frequent and direct patient interactions, focus on providing patient-centered care [4].

Historically, nurse-physician relationships were hierarchical, with doctors exercising authority and nurses expected to comply by focusing solely on patient care and following medical orders. This traditional dynamic portrayed the doctor as paternal and directive, while the nurse had a subordinate role. However, the increasing complexity of modern healthcare systems has driven a necessary shift toward a more collaborative model [5]. This evolving dynamic emphasizes shared responsibilities between nurses and physicians, where both parties work together to resolve issues and jointly create and implement patient care plans. Effective collaboration requires integrated decision-making authority, accountability, and power in managing patient care. Evidence of mutual trust, open communication, and respect is essential, with both parties valuing each other’s knowledge and opinions to foster a productive partnership [6].

Understanding how medical and nursing students perceive nurse-physician collaboration is critical for developing educational strategies that foster IPC from the outset of their careers. By exploring these perceptions, healthcare educators and policymakers can identify potential gaps in training and promote a culture of collaboration that can be carried into professional practice [7].

Furthermore, public opinion is a highly influential force in shaping social norms and values. Consequently, public perception can create a disparity between who people are and who they want to be [8]. So, various factors may shape students’ perceptions of a profession, including personal and community influences. Specifically, the image of nursing as a profession is shaped by public perception, societal norms, media portrayal, nurses’ behaviors, having a family member in the nursing field, interactions between nurses and physicians, exposure to workplace violence, and the risk of occupational health hazards. These interconnected elements collectively influence how nursing is perceived and valued within society [9].

Providing a shared educational environment where nursing and medical students interact makes their collaboration particularly noteworthy and shapes their professional relationships and teamwork in practice [10].

Although nurse-physician collaboration is crucial for enhancing healthcare outcomes, most studies primarily examine practicing healthcare professionals. This leaves a gap in understanding how future physicians and nurses view IPC during their education and training. Furthermore, there is limited research on the specific factors that contribute to positive perceptions among these students. Addressing this issue is particularly important for Egyptian students at Al-Azhar University, as their perspectives can inform strategies to improve IPC integration in medical and nursing curricula.

### So, the aim of the current study is

1. To explore the perceptions of medical and nursing students at Al-Azhar University regarding nurse-physician collaboration.
2. To identify the related factors of positive perceptions toward nurse-physician collaboration among medical and nursing students.

### The research questions are

1. How do medical and nursing students at Al-Azhar University perceive nurse-physician collaboration?
2. Which factors are related to positive perceptions of medical and nursing students toward nurse-physician collaboration?

## Participants and methods

### Study design and setting

A comparative cross-sectional study was conducted over eight months among students at Al-Azhar University in Cairo, Egypt.

**Study Population** included female medical and nursing students at Al-Azhar University at two stages:

#### Inclusion criteria

The study included female medical and nursing students at Al-Azhar University in two stages.

- *Educational Stage*: The study involved medical students in the fourth and fifth years as part of the clinical phase of the Integrated Learning Program at the Faculty of Medicine (for girls). Additionally, nursing students from the final two grades, either from the Nursing Institute at Al-Zahraa University Hospital or from the Faculty of Nursing (for girls), were recruited for the study.
- *Training Stage*: The study included house officers who had completed their medical education and had undergone two-year practical training, alongside internship nurses in their one-year training.

#### Exclusion criteria

The study excluded non-Egyptian students studying at the university and those who had not spent at least three months in training.

### Sampling technique

The study’s sample size was calculated using the Open Epi software https://www.openepi.com/SampleSize/SSCohort.htmassuming a 95% confidence level, an absolute precision of 5% (d = 0.05), and the estimated proportion of 50% for IPC with taking into consideration the size of the medical and nursing students through the academic year 2023–2024. The minimum sample size was 323, which was rolled up to 364 to account for a 10% non-response rate. The study allocated students proportionally when selecting medical and nursing students, considering whether they were in their educational phase or training. Then, a convenient sampling approach was used to achieve the required sample size. Students were recruited through institutional announcements and direct invitations, and participation was entirely voluntary, with informed consent obtained from all participants.

### Study tools and procedures

In this study, a structured, self-administered questionnaire took about 20 min to complete. It consisted of closed and open-ended questions to gather the following relevant data from students:

1. **Socio-demographic characteristics of students** included age, residence, parents’ level of education, and average family income.
2. **Academic data of students** included the study place, academic year, and history of clinical training in hospitals.

### Student perception toward Doctor-Nurse IPC

It was assessed using the validated Jefferson Scale of Attitudes Toward Physician–Nurse Collaboration (JSAPNC) [11], which was originally developed by Hojat et al. [12]. This 15-item tool is widely used across various settings, educational programs, and cross-cultural studies. It comprises four subscales: Shared Education and Teamwork (7 items), Caring as Opposed to Curing (3 items), Nurses’ Autonomy (3 items), and Physician’s Dominance (2 items).

Responses were measured on a 4-point Likert scale (1 = strongly disagree to 4 = strongly agree), with two negatively worded items reverse-scored. The total score ranged from 15 to 60, where higher scores indicated more positive attitudes toward IPC. Attitudes were classified as positive or negative based on the median score during analysis.

The Arabic version of the JSAPNC, psychometrically tested by Elsous et al. [13], demonstrated good validity and reliability (Cronbach’s alpha = 73.2, Pearson’s *r* = 0.79). The questionnaire was reevaluated in our setting for its reliability, obtaining Cronbach’s alpha of 0.8. A pilot test with 20 students (their responses not included in the analysis) ensured clarity and effectiveness, requiring no modifications.

4. **Factors related to students’ positive perceptions of collaboration include** previous personal experiences, the experiences of friends or relatives, participatory observations made during clinical training in health care, representations of collaboration between doctors and nurses on social media, and the availability of curricular content or supplementary courses focused on teamwork and communication skills.

The key factors related to students’ perceptions of IPC were identified based on a review of existing literature and adapted to the study context where students selected factors from a multiple-choice list, with the option to choose multiple responses. An open-ended question was also provided to allow them to specify any additional factors.

**Data management & Statistical analysis**. Data entry was completed, coded, and reviewed for potential errors before statistical analysis using the Statistical Package for the Social Sciences (SPSS), version 16.*IBM Corp. (2007). SPSS 16.0 Statistical Software. Chicago: SPSS Inc.* Quantitative data were assessed for normality, and accordingly, they were presented as either mean ± standard deviation (SD) for normally distributed data or median with interquartile range (IQR) for non-normally distributed data. Comparisons were conducted using the independent t-test for normally distributed quantitative data and the Mann-Whitney U test for non-normal distributions. Categorical variables were reported as frequency (N) and percentage (%), and Pearson’s chi-squared test (χ²) was utilized for categorical data comparisons. Results were presented in appropriate tables and graphs, with a p-value of less than 0.05was considered statistically significant.

## Results

Out of the 364 students studied, 153 were medical students, including 25 house officers, and 211 were nursing students, including 20 students in the internship phase.

Additional sociodemographic data of the total sample are presented in Table S1 of the supplementary file.

**Fig. 1:**
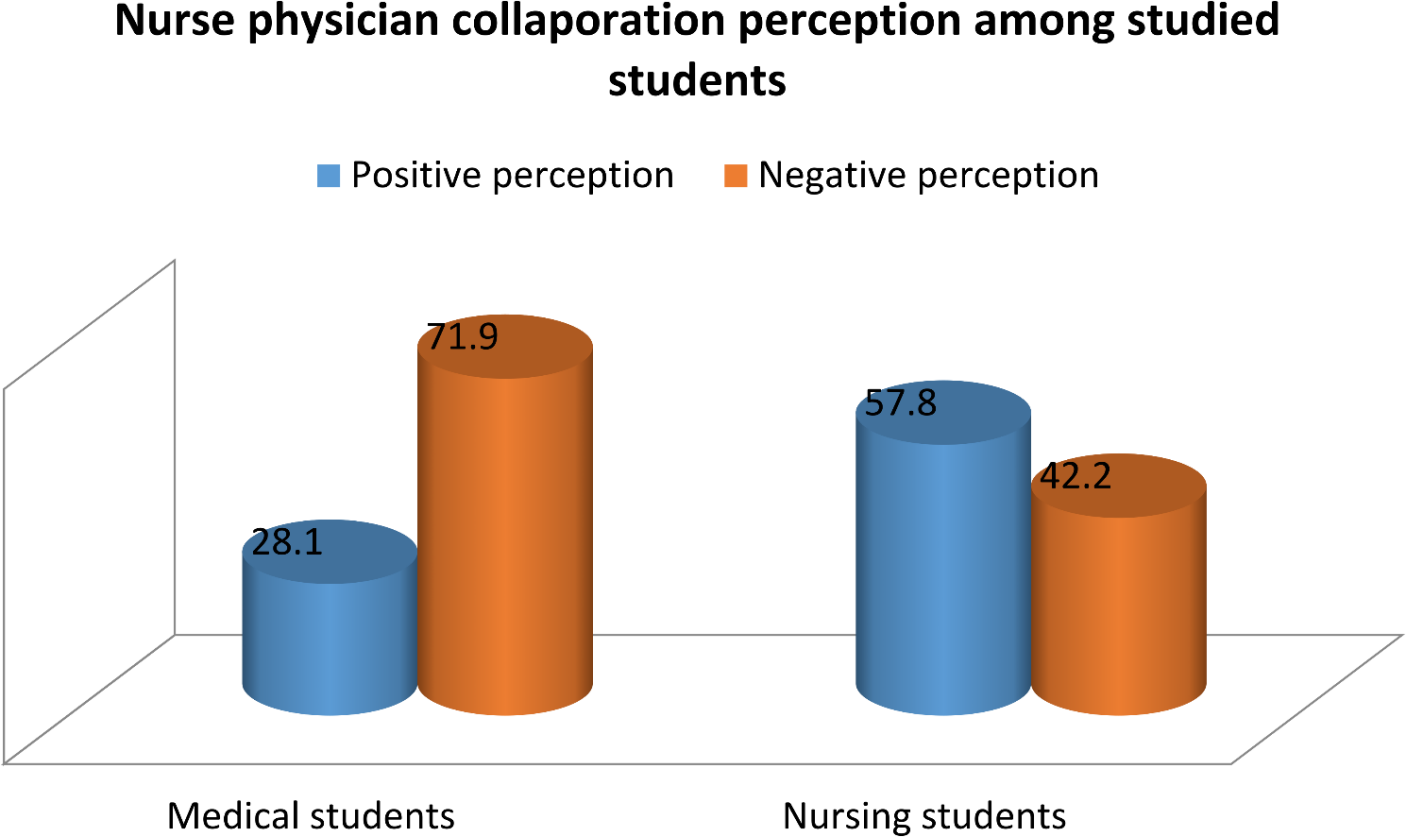
Perception toward collaboration among medical and nursing students

In assessing the attitudes of students toward Nurse-Physician Collaboration, nursing students scored higher on all subscales of the JSAPNC compared to medical students. Their mean scores were greater for shared education and teamwork (22.8 vs. 22.1), caring versus curing (9.9 vs. 8.5), nurse autonomy (9.9 vs. 9.4), and physician dominance (5.4 vs. 4.9). Overall, nursing students had a higher total score (47.9 vs. 44.8), indicating a more positive attitude. All differences were statistically significant, as shown in Table 1. The students’ attitudes were categorized as positive or negative based on the median of the overall JSAPNC score, as illustrated in Fig. 1. The results indicated that only 28.1% of medical students had a positive perception of nurse-physician collaboration, while a majority of nursing students (57.8%) viewed it positively. This difference is statistically significant (p-value < 0.001).

**Table 1:** Attitude of the studied medical and nursing students toward nurse physician collaboration

Domains of JSAPNC	Medical students (153)	Nursing students (211)	*p*-value
**Shared education and teamwork**			
Mean	22.1	22.8	0.00*
Median(IQR)	21 (20–24)	23(21–25)	
**Caring versus curing**			
Mean	8.5	9.9	0.01*
Median (IQR)	9 (7–9)	9(9–11)	
**Nurse autonomy**			
Mean	9.4	9.9	0.00*
Median(IQR)	9 (9–10)	10(9–11)	
**Physician dominance**			
Mean	4.9	5.4	0.00*
Median(IQR)	5 (4–6)	5(4–7)	
**Total score**			
Mean	44.8	47.9	0.00*
Median(IQR)	44 (42–47)	47 (44–51)	

* Mann–Whitney U

Examining the sociodemographic characteristics of students with positive perceptions in both groups revealed distinct differences. Among students with positive perceptions, the mean age was 19.8 years for nursing students and 21.9 years for medical students. A lower proportion of nursing students had parents with higher education levels, with only 32% of mothers and 45.1% of fathers having higher educational attainment, compared to 62.8% and 81.4% among medical students, respectively. Fewer nursing students resided in rural areas (28.7%) compared to medical students (51.2%). Regarding family income, 51.6% of nursing students reported having an adequate income, significantly lower than the 74.7% reported by medical students. All these differences were statistically significant (*p* < 0.05)as demonstrated in Table 2.

**Table 2:** Socio-demographic factors associated with positive perception among the studied medical and nursing students

Variables	Medical students (43)	Nursing students (122)	*p*-value
**Age of students: - Mean (SD)**	21.9 (1.3)	19.8 (1.5)	0.00**
**Mother educational level: No (%)**			
Low(secondary and below)	16 (37.2)	83 (68.0)	0.00*
High(above secondary)	27(62.8)	39 (32.0)	
**Father educational level: No (%)**			
Low(secondary and below)	8 (18.6)	67 (54.9)	0.00*
High(above secondary)	35(81.4)	55(45.1)	
**Residence: No. (%)**			
Rural	22 (51.2)	35(28.7)	0.01*
Urban	21 (48.8)	87 (71.3)	
**Family income: No. (%)**			
Inadequate	11 (25.6)	59 (48.4)	0.01*
Adequate	32 (74.4)	46 (51.6)	

* Pearson Chi-Square (*X*^*2*^*)* test, ** Independent sample *t-*test

Additionally, investigating academic and clinical factors among students with positive perception revealed that 51.2% of medical students attended inter-professional skills courses compared to 32% of nursing students. Furthermore, 81.4% of medical students and 87.7% of nursing students reported that communication and inter-professional skills were included in their curriculum; this difference was not statistically significant (p-value = 0.3). A significant difference was observed in clinical training experience, with only 9.3% of medical students having this experience compared to 55.7% of nursing students (*p* < 0.001). The median duration of the experience was shorter for medical students (2 years) than for nursing students (3 years), with no significant difference (p-value = 0.7)as indicated in Table 3.

**Table 3:** Academic and clinical factors associated with positive perception among the studied medical and nursing students

Variables	Medical students (43)	Nursing students (122)	*p*-value
Attending extracurricular courses about inter-professional skills No. (%)			
No	21 (48.8)	83 (68.0)	0.03*
Yes	22(51.2)	39 (32.0)	
**Curriculum content of communication and inter-professional skills: No (%)**			
No	8 (18.6)	15 (12.3)	0.3*
Yes	35(81.4)	107 (87.7)	
**History of clinical training in hospitals: No. (%)**			
No	39(90.7)	54(44.3)	0.000*
Yes	4 (9.3)	68(55.7)	
**Duration of experience**: - Median (IQR)	2 (2–4)	3 (3–4)	0.7**

* Pearson Chi-Square (*X*^*2*^*)* test, ** Mann–Whitney U.test

**Fig. 2:**
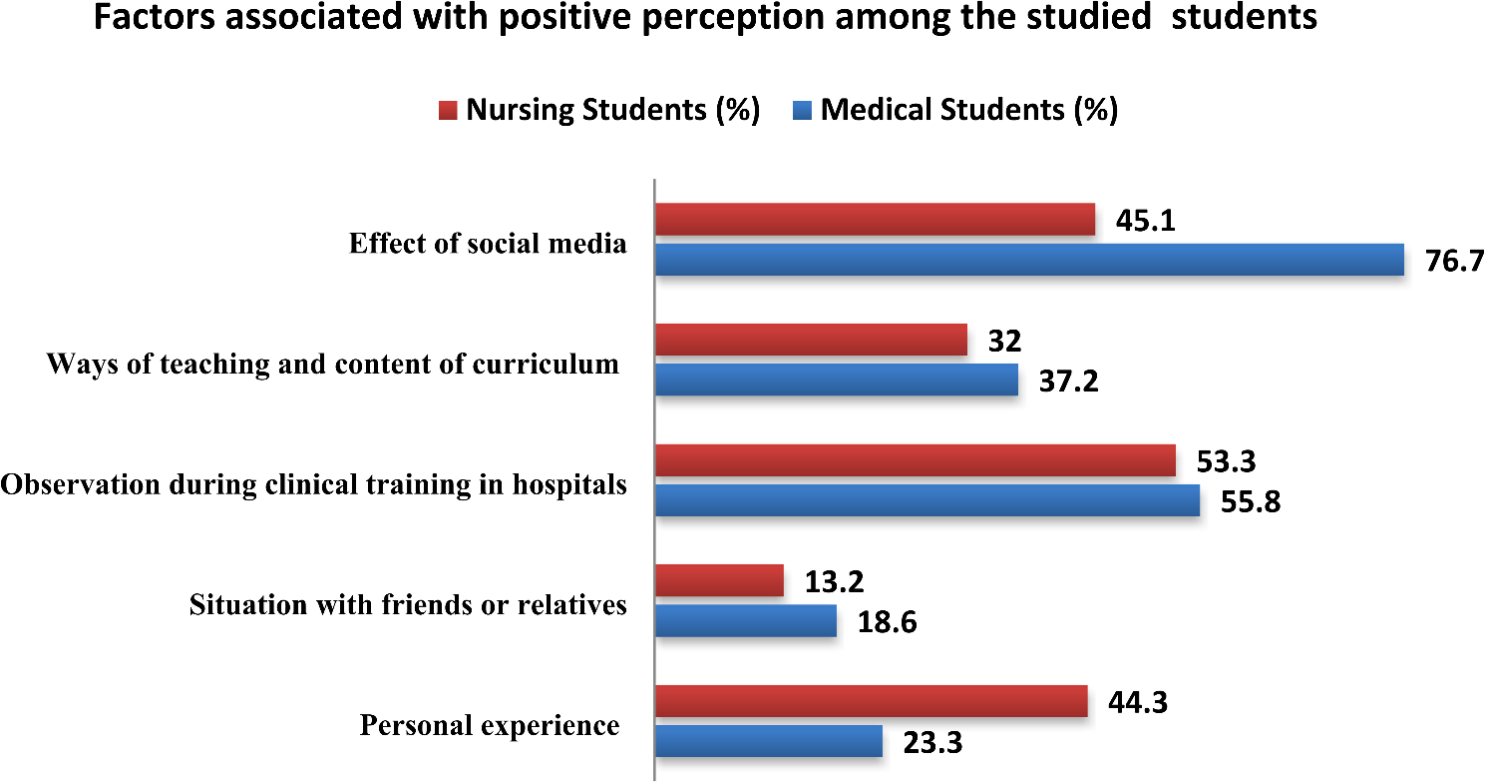
Self-reported factors associated with positive perception among the studied medical and nursing students

In terms of factors shaping a *positive attitude* from students’ perspectives, nearly half (53.3%) of nursing students identified observation in clinical hospital settings as a key determinant, followed by personal experience. Meanwhile, 76.7% of medical students highlighted the role of social media in shaping their positive perceptions, with clinical training observations as a secondary factor, as presented in Fig. 2.

A comparison of students in different phases of their education across various JSAPNC domains shows that medical students have notably higher median scores in physician dominance (5) and total score (44) than house officers, who scored 4 and 42, respectively. However, both groups show no significant differences in areas such as shared education and teamwork, caring versus curing, and nurse autonomy. In contrast, there are no significant differences between nursing students and internship nurses across all subdomains and the JSAPNC total score (p-value > 0.05)as outlined in Table 4.

**Table 4:** Comparison between different groups of medical and nursing students regarding interprofessional collaboration perception

Domains Groups	Shared Education and Teamwork median(IQR)	Caring versus Curing median(IQR)	Nurse Autonomy median(IQR)	Physician Dominance median(IQR)	Total score
**Medical Students (128)**	21.5 (20–24)	9 (8–9)	9 (9–10)	5 (4–6)	44(42-47.8)
**Medical House Officers (25)**	21 (20–23)	8 (7–9)	9 (9–10)	4 (4–5)	42(40.5–46)
**p-value**	0.3*	0.1*	0.9*	0.001*	0.05*
**Nursing Students (191)**	23 (21–25)	9 (9–11)	10 (9–11)	5 (4–7)	48(44–52)
**Internship Nurses (20)**	23 (21–25)	9 (9-10.75)	10 (9–10)	5 (4–6)	45(44-50.8)
**p-value**	0.9*	0.4*	0.4*	0.2*	0.4*
**Nursing Faculty Students (73)**	24 (21–27)	10 (9–12)	10 (9–11)	6 (5–7)	50(46–56)
**Nursing School Students (138)**	22 (21–24)	9 (9–10)	10 (9–12)	5 (4–6)	47(44–50)
**p-value**	0.000*	0.002*	0.001*	0.000*	0.000*

*Mann–Whitney U.test

In comparing nursing students based on their study level (either school or faculty) across various JSAPNC domains as shown in Table 4, the median scores for the total score and all domains were significantly higher among nursing faculty students than nursing institute students, except for nurse autonomy, which was equal between the two groups. All observed differences were statistically significant (p-value < 0.01).

## Discussion

Medical and nursing students’ attitudes toward IPC are crucial for fostering effective teamwork in healthcare settings and can influence their future professional interactions and patient care outcomes. This study aimed to explore students’ perceptions and investigate the various related factors.

The current findings indicate that nursing students demonstrated more favorable attitudes and scored higher in all subscales of the JSAPNC. This observation aligns with various studies that highlight the complexities of IPC in healthcare environments with varied attitudes. Park et al. [14] reported generally positive attitudes toward collaboration in healthcare settings, with nursing students exhibiting more favorable attitudes than their medical counterparts. Also, Wang et al. [15] demonstrated that nursing students achieved statistically significant higher scores in attitudes toward collaboration. Additionally, Boonmaket al. [16] explored the perceptions of IPC among final-year health science students in Thailand, in which nursing students demonstrated the highest IPC perceptions, while medical and public health students scored the lowest. Conversely, Ardahan et al. [17] found that medical students scored significantly higher than nursing students on measures of collaborative attitudes.

To investigate the discrepancies in perceptions among medical and nursing students, we will focus on factors contributing to positive perceptions.

When comparing students with positive attitudes in both groups, we identified significant *socio-demographic differences as nursing* students had parents with lower education levels and had lower-income families compared to medical students. This can be explained by Social Cognitive Career Theory(SCCT) [18], which suggests that individuals from challenging backgrounds develop stronger self-efficacy and resilience and strive to overcome barriers. For these nursing students, IPC may be viewed as a crucial opportunity to develop their professional networks, become more competent, and progress in their professions.

Also, the current study sheds light on *academic and clinical factors* associated with the positive perception among students, noting that a greater proportion of nursing students reported having communication and interprofessional skills included in their curriculum compared to their medical counterparts. While this difference is not statistically significant, it underscores the value of incorporating communication skills training into educational programs to enhance students’ appreciation for teamwork. This result is consistent with Zakerimoghadam et al. study [19] which emphasized the importance of IPE in developing skilled nurses and enhancing patient safety. Likewise, Homeyer et al. [20] pointed out the beneficial effects of IPE on teamwork and collaboration while acknowledging the challenges in aligning curricula between medical and nursing programs.

Furthermore, the current study revealed that 55.7% of positive attitude nursing students significantly had clinical training compared to 9.3% of medical students. However, a study done by Dahlawi et al. [21] found no significant difference in the medical students’ attitudes about collaboration when the results were broken down according to clinical exposure. Typically, students who are involved in multidisciplinary teams often develop a more collaborative mindset through their training and practical experiences, which can result in more positive attitudes [22].

According to Ferrara et al. [23], the blend of a comprehensive communication curriculum and extensive clinical experience enhances nursing students’ attitudes toward collaboration.

However, by analyzing the *self-reported factors of perception* among positively perceived students, nearly half (53.3%) of nursing students identified observation in clinical hospital settings as a key determinant of their positive perception. This aligns with recent findings by Hooven [24], which reported that 55.8% of nursing students attributed their favorable attitude to witnessing collaboration in clinical hospital settings, further justifying the role of clinical exposure in shaping positive perceptions and underscoring the significant role of teaching faculty in shaping students’ perceptions of IPC through role modeling, feedback, training, and clinical exposure.

While medical students indicated that social media is the main factor for having less positive collaborative perception compared to nursing students, this can be attributed that social media may play a role in shaping professional expectations, often reinforcing existing stereotypes regarding the nursing profession. This is supported by Ventola [25], who highlighted that social media use in healthcare is associated with risks such as the spread of misinformation, breaches of confidentiality, and unprofessional behavior. Although nurses actively use platforms like Twitter and Facebook to advocate for their profession and challenge stereotypes, the notion of physician superiority persists due to longstanding media portrayals and societal biases [26]. In the Egyptian healthcare context, physicians view the nursing profession negatively, perceiving nurses as subservient and dependent decision-makers who merely execute physicians’ orders. Despite the introduction of university-level nursing education, the public image of nursing has not significantly improved [27].

Moreover, the present study gives insights into *the precipitation of collaboration among different phases of medical and nursing education*. We observed that medical students, compared to house officers, had a significantly overall positive attitude toward nurse-physician collaboration, although they scored higher in physician dominance. This difference may stem from medical students’ exposure to IPE, which fosters collaborative attitudes, while house officers face increased stress and responsibilities that might negatively impact their view of teamwork. As regards the perception of physician dominance, Belrhit et al. [28] revealed that higher dominance observed among medical students may indicate the hierarchical dynamics inherent in medical education where students perceive physicians as the primary leaders within the healthcare system.

When comparing nursing students to nursing internships, no significant differences were observed across all domains of the JSAPNC. In contrast, Hossny et al. [29] observed that internship nurses are not satisfied with the level of collaboration between nurses and physicians compared to physicians and staff nurses. The stability in attitude in both studied groups suggests that they may receive similar training and exposure to the principles of IPC regardless of their educational level.

Further comparison revealed that nursing faculty students had a more positive attitude of IPC than nursing institute students, which can be attributed to their gaining a more comprehensive education; as their education progresses, they come to value interprofessional teamwork, which in turn leads to more positive attitudes.

### Strengths and limitations

This study’s strengths include the use of a validated tool and the inclusion of medical and nursing students at different educational and learning stages. However, this study was conducted at a single institution within faculties for girls only, so its generalizability may be restricted. Moreover, the cross-sectional design limits the ability to track changes in perception over time, emphasizing the need for longitudinal studies. Additionally, focusing solely on final year students constrains comparisons across academic levels. Another limitation is the potential for response bias, as extreme attitudes may have affected the findings. To address these issues, future research should expand the sample size and include multiple institutions to enhance applicability and minimize potential biases.

### Practical implications

Considering the current study findings, educators should enhance IPE by integrating communication and teamwork training into medical curricula while serving as role models for positive nurse-physician collaboration. Policymakers should support curriculum reforms that introduce early collaborative clinical experiences for both medical and nursing students. Clinical training programs should implement structured interdisciplinary training to strengthen teamwork skills and address perception gaps. Given the influence of social media on medical students, targeted interventions such as educational campaigns, digital literacy training, and interactive online IPE modules can help foster more positive and informed attitudes toward IPC.

## Conclusion

Nursing students generally exhibited a more positive attitude toward nurse-physician IPC compared to medical students. Nursing students emphasized their clinical training observation as a key role, whereas medical students relied more on social media to shape their perceptions. Additionally, medical students demonstrated a more favorable perception of IPC than house officers, with no significant differences observed in attitudes between nursing students and internship nurses.

## Electronic supplementary material

Below is the link to the electronic supplementary material.

Supplementary Material 1: Sociodemographic data of the total sample are presented in Table S1 as a supplementary file.

## Data Availability

Data is provided within the manuscript or supplementary information files.
