# Supplementary material for: Perception of medical and nursing students towards interprofessional nurse-physician collaboration at Al-Azhar University: a comparative cross-sectional study
Source: BMC Med Educ. 2025 May 5;25:651. doi: 10.1186/s12909-025-07150-6 (PMC12054296; doi:10.1186/s12909-025-07150-6)
Supplement: Supplementary file 1 — Supplementary Material 1: Sociodemographic data of the total sample are presented in Table S1 as a supplementary file. [file 12909_2025_7150_MOESM1_ESM.docx]

**Table S1:** Socio-demographic characteristics among the studied medical and nursing students

| **Variables** | **Medical students (153)** | **Nursing students**  **(211)** | **Significant Value** |
| --- | --- | --- | --- |
| **Age of students: - Mean (SD)** | 22.3 (1.2) | 19.7 (1.4) | p. value = 0.000** |
| **Mother educational level: No (%)** | | | |
| - Low(secondary and below) | 52 (34.0) | 149 (70.6) | p. value = **0.000*** |
| - High(above secondary) | 1. **(66.0)** | 62 (29.4) |  |
| **Father educational level: No (%)** | | | |
| - Low(secondary and below) | 32 (20.9) | 121 (57.3) | p. value = **0.000*** |
| - High(above secondary) | 1. **(79.1)** | 90(42.7) |  |
| **Residence: No. (%)** | | | |
| - Rural | 67 (43.8) | 49(23.2) | p. value= **0.000*** |
| - Urban | 86 **(56.2)** | 162 (76.8) |  |
| **Family income: No. (%)** | | | |
| -  Inadequate | 8 (5.2) | 31 (14.7) | p. value = **0.000*** |
| -  Just meet basic requirements | 37 (24.2) | 85 (40.3) |  |
| -  Meat basic requirements | 76 (49.7) | 80 (37.9) |  |
| - Able to save and invest money | 32 **(20.9)** | 15 (7.1) |  |

* Pearson Chi -Square (*X^2^)* test, **** Independent sample *t* test,

As shown in **Table** **S1**, the average age of medical students was 22.3 years, while nursing students averaged 19.7 years. Among medical students, 66.0% of their mothers and 79.1% of their fathers had higher education, compared to 29.4% of mothers and 42.7% of fathers of nursing students. Approximately 56.2% of medical students and 76.8% of nursing students lived in urban areas. Furthermore, 20.9% of medical students reported having financial savings or investments, compared to just 7.1% of nursing students. All differences were statistically significant (p-value <0.001).
